# COVID-19 vaccine hesitancy and its drivers among dental students at University of the Western Cape, South Africa

**DOI:** 10.4102/hsag.v27i0.1950

**Published:** 2022-10-20

**Authors:** Nicoline Potgieter, Faheema Kimmie-Dhansay, Ané Meyer, Savannah Marais, Ismail Mansoor, Yonela Mkololo, Masingita Maakana, Sisipho Mhlongo, Sinenhlanhla Makhoba, Shalom Mhlanga

**Affiliations:** 1 Department of Paediatric Dentistry, Faculty of Dentistry, University of the Western Cape, Cape Town, South Africa; 2 Department of Community Oral Health, Faculty of Dentistry, University of the Western Cape, Cape Town, South Africa

**Keywords:** COVID-19 vaccine hesitancy, prevalence, COVID-19 vaccine, dental students, academic institution

## Abstract

**Background:**

Vaccine hesitancy has seen an uprising over the decades, even though there have been many advances regarding vaccine-preventable diseases. Of late, vaccine hesitancy has resurged towards the coronavirus disease 2019 (COVID-19) vaccine. The SARS-CoV-2 virus has major effects on the human body and has led to the development of different vaccines, which have been shown to provide immunity against the novel coronavirus. Dentists are at an increased risk to COVID-19 because of the nature of their work. It is imperative to have high vaccination coverage for this group.

**Aim:**

The aim of this study is to determine vaccine hesitancy and drivers associated with vaccine hesitancy among dental students at a university in South Africa.

**Setting:**

A dental school in South Africa was chosen as the setting for this study.

**Methods:**

An analytical cross-sectional study was conducted by means of an anonymous, online, validated questionnaire to determine vaccine hesitancy.

**Results:**

Of the 205 dental students participated, 83.9% (*n* = 172) students were vaccine not hesitant. The main concerns regarding the vaccines were identified as safety and efficacy of the vaccine. Pressure by family or friends and the university to get vaccinated was evident.

**Conclusions:**

Vaccine hesitancy is high despite mandatory vaccination policies in South Africa. Specific drivers contributing to vaccine hesitancy were identified as doubt in the efficacy and safety of the vaccine.

**Contribution:**

This study has highlighted the prevalence of vaccine hesitancy among dental students at University of the Western Cape, prior to compulsory vacccination implementations.

## Introduction

According to MacDonald (2015), vaccine hesitancy is defined as ‘delay in acceptance or refusal of vaccination despite availability of vaccination services’. In a study by Kelekar et al. (2021) in the United States of America (USA), there was a 45% vaccine hesitancy among dental students, with a global vaccine hesitancy among dental students reported to be 22.5% (Riad et al. 2021). In Palestine, the vaccine hesitancy among dental students was determined to be 27% (Kateeb et al. 2021). As of March 2022, the coronavirus disease 2019 (COVID-19) pandemic has had a considerable disease burden globally (482 million) and in South Africa (3.71 million) (Dong, Du & Gardner 2020, WHO 2022). The mortality rate has reached up to 6.13 million globally and there have been almost 100 000 cases in South Africa (Dong et al. 2020). Dentists have been affected by the manner in which they can perform their work, as only essential care was allowed when COVID-19 regulations were first rolled out (Beier 2020).

Nations worldwide are trying to reduce the spread of the COVID-19 virus by enforcing different preventative measures such as physical distancing and wearing of face coverings as well as lockdown and travel restrictions. Regarding the latest omicron variant, there has been a reduction in COVID-19-related deaths and morbidity in South Africa (Kew & Sguazzin 2022) and globally. The impact of this has led to many countries lifting their lockdowns or even recommending the complete removal of mask-wearing laws (Keaton 2022). Immunity will remain the only preventative measure in place if all other protective measures are stopped. As of 29 March 2022, 11.2 billion vaccine doses have been administered globally (Ritchie et al. 2020).

Dentistry is categorised as a high-risk profession because of the nature of the work and aerosol-generating procedures. The proximity to patients as well as the possibility of treating an asymptomatic COVID-19 patient also contribute to an extended risk of contracting and even spreading the virus (Gamio 2020). Hence, both dentists and dental students in training are encouraged to be vaccinated (Kelekar et al. 2021). The COVID-19 vaccine, like any other vaccine, has to be accepted by a large number of the students for it to be effective and establish herd immunity (Pogue et al. 2020); however, according to Grech, Souness and Agius (2021), dentistry students showed more hesitancy towards the COVID-19 vaccine in comparison to medical students. In an Austrian study, vaccine hesitancy was driven by participants’ mistrust in the government (Riad et al. 2021; Schernhammer et al. 2022). Other drivers to vaccine hesitancy that were identified in dental undergraduate students in multiple upper- and low-income countries as insufficient knowledge about vaccines and mistrust in the pharmaceutical industry (Riad et al. 2021). According to the authors’ knowledge, there is currently no available literature on vaccine hesitancy among dental students in Africa.

## Research methods and design

The aim of this study, therefore, was to determine the vaccine hesitancy of dental students at the University of the Western Cape (UWC) Oral Health Centres. The secondary aim is to determine the drivers associated with vaccine hesitancy in this population.

An analytical cross-sectional study was conducted by means of an online self-administered questionnaire. A cross-sectional study is cost effective and easy to conduct and thus it was the preferred method of choice for this study (Sedgwick 2014). All registered undergraduate dental students (*n* = 416) were included in this study and recruited from September 2021 to October 2021. A convenience sampling technique was utilised to recruit participants, as this sampling technique is simple and cost effective (Sedgwick 2013). The intended sample size was calculated to be 200:


$$\begin{array}{l} m=\frac{{\left(Z\alpha /2\right)}^{2}p(1-p)}{d^{2}}=\frac{{\left(1.96\right)}^{2}\times 0.5(0.5)}{0.05^{2}}=384.16\\ n=\frac{m}{1+\frac{m-1}{N}}=\frac{384.16}{1+\frac{384.16-}{416}}=199.97 \end{array}$$ [Eqn 1]

A human resources representative e-mailed the sample population twice, one week apart. The e-mail contained the link with information about the study. Informed consent was obtained electronically before proceeding to the survey. Participation in the study was voluntary, and participants could withdraw from the study at any stage. Google Forms was used for data capturing, which was transferred to Microsoft Excel for data processing and analysis. The Excel file was imported into Stata software (StataCorp. 2019) by a qualified statistician who was blinded to the participants. Nominal data were described as frequencies and percentages, and the first-order analysis was conducted using a chi-square test or Fisher’s exact test, where appropriate. An unadjusted and adjusted logistic regression was used to determine whether there was an association between the various drivers and vaccine hesitancy. All tests were deemed statistically significant at *p* < 0.05.

### Ethical considerations

The study was approved by the research committee at the Dental Faculty, and ethical clearance was obtained from the UWC Biomedical Research Ethics Committee, reference number: BM21/6/16. All study participants had given informed consent before taking part in this study.

## Results

A total of 49% UWC dentistry students (205 of the 416), participated in this study. Thirty-three participants (16.1%) were vaccine hesitant. Among the participants, there were no statistically significant associations between vaccine hesitancy and demographics, including the year of study, age or sex (Table 1). Internal validity was measured at 0.864 using Cronbach’s alpha. There was a statistical significant association (*p* < 0.001) between vaccine hesitancy and whether the participants felt that vaccines were generally effective (Table 1).

**TABLE 1:** Responses to close-ended questions related to the coronavirus disease 2019 vaccine.

Drivers associated with COVID-19 vaccine hesitancy	Total		Vaccine hesitant				*p*
	*n*	%	Yes (*n* = 33)		No (*n* = 172)		
			*n*	%	*n*	%	
**Year of study**							
BDS I	54	26.3	11	20.4	43	79.6	0.167
BDS II	36	17.6	9	25.0	27	75.0	
BDS III	40	19.5	5	12.5	35	87.5	
BDS IV	45	21.9	3	6.7	42	93.3	
BDS V	30	14.6	5	16.7	25	83.3	
**Age**							
< 20 years	53	25.9	9	16.9	44	83.0	0.948
20–21 years	86	41.9	13	15.1	73	84.9	
≥ 22 years	66	32.2	11	16.7	55	83.3	
**Sex**							
Female	142	69.3	22	15.5	120	84.5	0.724
Male	63	30.7	11	17.5	52	82.5	
**Have you ever received a vaccine in your lifetime?**							
No	7	3.4	3	42.9	4	57.1	0.084
Yes	198	96.6	30	15.2	168	84.9	
**Do you believe that vaccines are generally effective?**							
No or unsure	16	7.8	10	62.5	6	37.5	< 0.001*
Yes	189	92.2	23	87.8	166	12.2	
**How would you describe your knowledge on the COVID-19 vaccine?**							
Average	103	50.2	20	19.4	83	80.6	0.09
Excellent	9	4.4	1	11.1	8	88.9	
Good	76	37.1	7	9.2	69	90.8	
Poor or very poor	17	8.3	5	29.4	12	70.6	
**Do you understand the concept of herd immunity?**							
No	57	27.8	15	26.3	42	73.7	
Yes	148	72.2	18	12.2	130	87.8	0.013*
**Do you believe that the COVID-19 vaccine can prevent COVID-19 infection?**							
No	60	29.3	23	38.3	37	61.7	< 0.001*
Yes	145	70.7	10	6.9	135	93.1	
**Is the COVID-19 vaccine safe?**							
No or unsure	83	40.5	31	37.4	52	62.7	< 0.001*
Yes	122	59.5	2	1.6	120	98.4	
**Is the vaccine necessary to combat the global coronavirus pandemic?**							
No or unsure	47	22.9	24	51.1	23	48.9	< 0.001*
Yes	158	77.1	9	5.7	149	94.3	
**Will you recommend that family and friends take the COVID-19 vaccine?**							
No	38	18.5	25	65.8	13	34.2	< 0.001*
Yes	167	81.5	8	4.8	159	95.2	
**Do you have any doubts regarding the COVID-19 vaccine?**							
No	90	43.9	1	1.1	89	98.9	< 0.001*
Yes	115	56.1	32	27.8	83	72.2	
**Do you believe that face coverings, sanitation and physical distancing will not be necessary once you’ve received the COVID-19 vaccine?**							
No	181	88.3	30	16.6	151	83.4	0.773
Yes	24	11.7	3	12.5	21	87.5	

COVID-19, coronavirus disease 2019; BDS, bachelor of dental surgery.

* , Statistically significant.

**FIGURE 1:**
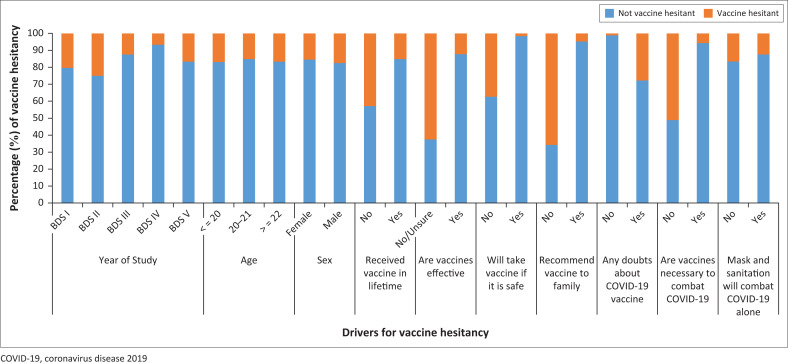
Vaccine hesitancy by various drivers.

A majority of the participants did not have vaccine hesitancy (Figure 1). There was a statistically significant association between vaccine hesitancy and whether the participants had any doubts regarding the COVID-19 vaccine, whether they would recommend the COVID-19 vaccine to family or friends and whether the dental students believed that the COVID-19 vaccine can prevent COVID-19 infection (*p* < 0.001) (Table 1).

Half of the students’ self-reported level of knowledge regarding the COVID-19 vaccine was ‘average’ (50.2%, *n* = 103) with only 4.4% (*n* = 9) of students admitting ‘excellent’ knowledge (Table 1). The three main sources reported by the student for obtaining knowledge were ‘healthcare workers’ (*n* = 125), ‘health officials’ (*n* = 125) and ‘social media’ (*n* = 122). Although 77.1% (*n* = 158) of participants believed that the vaccine is necessary to combat the pandemic, 27.8% (*n* = 57) of participants did not fully understand the concept of herd immunity (Table 1).

There was no association between vaccine hesitancy and history of any vaccination, knowledge of COVID-19 vaccination and belief that no more face coverings, sanitation and physical distancing will prevent COVID-19, respectively (*p* > 0.05) (Table 1).

Most students (*n* = 140) did not feel pressured to get vaccinated; however, the pressure felt from the university (*n* = 46) followed by family and friends were reported (*n* = 39). Almost 45% (*n* = 90) of participants said that they have doubts regarding the COVID-19 vaccine, with concerns regarding the safety (*n* = 97) of the vaccine being the major contributing factor. Concerns regarding the efficacy (*n* = 67) and mistrust in the development of the vaccines (*n* = 65) were also evident. Interestingly, dental students having doubts regarding the vaccine, 81.5% (*n* = 167) would recommend the vaccine to their family and friends.

In a multivariate logistic regression, participants who felt unsure that the COVID-19 vaccine was safe had a 9.92 (1.9–53.6) adjusted odds of experiencing vaccine hesitancy, *p* = 0.008 (Table 2). In addition, participants who were not likely to recommend the vaccine to family and friends had an increased odds of 26.2 (7.8–87.5), *p* < 0.001, of experiencing vaccine hesitancy (Table 2). Furthermore, participants who had any doubts regarding the COVID-19 vaccine and those who did not believe that face coverings and sanitation and physical distancing will prevent the spread of COVID-19 had an increased odds of 12.62 (1.3–125.8), *p* = 0.031 and 6.76 (1.2–38.5), *p* = 0.031, of experiencing vaccine hesitancy compared to participants who did not experience vaccine hesitancy, respectively (Table 2).

**TABLE 2:** Multivariate logistic regression analysis of close-ended questions.

Drivers associated with COVID-19 vaccine hesitancy	Undjusted			Adjusted		
	Odds ratio	95% CI	*p*	Odds ratio	95% CI	*p*
**Age**						
< 20	-	-	-	-	-	-
20–21 years old	0.87	0.3–2.0	0.77	-	-	-
≥ 22	0.98	0.4–2.6	0.964	-	-	-
**Sex**						
Female	-	-	-	-	-	-
Male	1.15	0.5–2.6	0.724	-	-	-
**Year of study**						
BDS I	1.3	0.5–3.6	0.605	-	-	-
BDS II	0.56	0.2–1.8	0.32	-	-	-
BDS III	0.28	0.1–1.1	0.063	-	-	-
BDS IV	0.78	0.2–2.5	0.679	-	-	-
BDS V	-	-	-	-	-	-
**Do you believe that vaccines are generally effective?**						
No or unsure	-	-	-	-	-	-
Yes	0.08	0.03–0.3	< 0.001*	-	-	-
**Have you ever received a vaccine in your lifetime?**						
No	-	-	-	-	-	-
Yes	0.24	0.1–1.1	0.069	-	-	-
**How would you describe your knowledge on the COVID-19 vaccine?**						
Average	-	-	-	-	-	-
Excellent	0.52	0.1–4.4	0.547	-	-	-
Good	0.42	0.2–1.1	0.065	-	-	-
Poor or very poor	1.73	0.6–5.5	0.351	-	-	-
**Do you understand the concept of herd immunity?**						
No	-	-	-	-	-	-
Yes	0.39	0.2–0.9	0.016*	-	-	-
**Do you believe that the COVID-19 vaccine can prevent COVID-19 infection?**						
No	-	-	-	-	-	-
Yes	0.12	0.1–0.3	< 0.001*	-	-	-
**Is the COVID-19 vaccine safe?**						
No or unsure	0.03	0.01–0.1	< 0.001*	9.92	1.8–53.6	0.008*
**Is the vaccine is necessary to combat the global coronavirus pandemic?**						
No or unsure	-	-	-	-	-	-
Yes	0.06	0.02–0.2	< 0.001*	-	-	-
**Will you recommend that family and friends take the COVID-19 vaccine?**						
Yes	0.03	0.01–0.1	< 0.001*	26.2	7.8–87.5	< 0.001*
No	-	-	-	-	-	-
**Do you have any doubts regarding the COVID-19 vaccine?**						
No	-	-	-	-	-	-
Yes	34.31	4.6–256.8	0.001*	12.62	1.3–125.8	0.031*
**Do you believe that no more face coverings, sanitation and physical distancing will be necessary once you’ve received the COVID-19 vaccine?**						
Yes	0.72	0.2–2.6	0.611	6.76	1.2–38.5	0.031*
No	-	-	-	-	-	-

COVID-19, coronavirus disease 2019; CI, confidence interval

* , Statistically significant.

## Discussion

There is a very high rate of vaccine hesitancy (16.1%) prevalence in this population. Among the UWC’s dental students, 29.3% do not believe that the vaccines available can prevent COVID-19 and its associated complications, which is similar to a study conducted among 248 dental students from three schools, which reported that 54% of dental students were concerned that the vaccine may not be effective (Mascarenhas et al. 2021).

A study among South Carolina college students reported that students primarliy trust scientists, followed by healthcare providers and then health authorities (El-Elimat et al. 2021). This was consistent with the results obtained from the UWC’s dental students, where most participants relied on their healthcare workers such as doctors and health officials to obtain information relating to vaccines.

Social media was also identified as a primary source of knowledge (*n* = 122), which is applicable to the age of the sample population. Information on social media is, however, not controlled and may provide misinformation, driven by spectator value and excitement, contributing to doubt and uncertainties among students. A study that was done in France indicated that vaccine acceptance and practices were better when information is obtained from healthcare providers as opposed to receiving information from relatives or from the Internet (Charron, Gautier & Jestin 2020).

A study done by Kelekar et al. (2021) conducted in three U.S. dental schools involving 248 students investigated the willingness of dental students to accept the vaccine. Themes identified in dental students’ negative responses included vaccine safety and efficacy, the vaccine’s rapid development and implementation, politicisation, trust in regulatory agencies, resources and the education of the public. These concerns were also evident among the UWC students, who also identified safety, efficacy and mistrust in the development of the vaccine as the major reasons for doubting the vaccine. It is pivotal to propagate accurate and transparent information about the safety and efficacy of vaccines in order to gain the population’s trust, especially those who are sceptical and hesitant (Siegrist & Zingg 2014).

According to the authors’ knowledge this type of study has not been conducted before in Africa. A limitation of this study is the small sample size. This could be because of non-response bias, as vaccine-hesitant participants would be less likely to participate.

## Conclusion

Vaccine hesitancy among undergraduate dental students at this healthcare facility is astonishingly high. Students still expressed doubts regarding the efficacy, safety, long-term side effects and effectiveness of the vaccines. Further research should be conducted to determine the feelings of students and to record any side effects experienced by dental students, after mandatory vaccination implementation on dental students in South Africa.
